# Green wastewater treatment of repurposed COVID-19 therapy (levofloxacin) using synthesized magnetite pectin nanoparticles, comparison with mesoporous silica nanoparticles

**DOI:** 10.1186/s13065-023-01048-4

**Published:** 2023-10-09

**Authors:** Christine M. El-Maraghy, Sarah S. Saleh, Mervat S. Ibrahim, Omnia A. El-Naem

**Affiliations:** 1 Analytical Chemistry Department, Faculty of Pharmacy, October University for Modern Sciences and Arts (MSA), 6th October City, 11787 Egypt; 2 Pharmaceutics Department, Faculty of Pharmacy, October University for Modern Sciences and Arts (MSA), 6th October City, 11787 Egypt

**Keywords:** Fluoroquinolones, Adsorption, Hexagon, AGREE, Levofloxacin

## Abstract

**Rationale:**

Antibiotics have been detected worldwide in the aquatic environment. Moreover, certain classes of antibiotics have been repurposed for the management of COVID-19, which increased their use and presence in wastewater. Their occurrence even in low concentrations leads to the development of antibiotic resistance.

**Methodology:**

Magnetite pectin nanoparticles (MPNP) were fabricated and compared to an established model of mesoporous silica nanoparticles (MSNP). Our studied adsorbate is levofloxacin, a fluoroquinolone antibiotic, commonly used in managing COVID-19 cases.

**Results:**

The influence of various factors affecting the adsorption process was studied, such as pH, the type and concentration of the adsorbent, contact time, and drug concentration. The results illustrated that the optimum adsorption capacity for antibiotic clearance from wastewater using MPNP was at pH 4 with a contact time of 4 h; while using MSNP, it was found to be optimum at pH 7 with a contact time of 12 h at concentrations of 10 µg/mL and 16 g/L of the drug and nanoparticles, respectively, showing adsorption percentages of 96.55% and 98.89%. Drug adsorption equilibrium data obeyed the Sips isotherm model.

**Discussion and conclusion:**

HPLC assay method was developed and validated. The experimental results revealed that the MPNP was as efficient as MSNP for removing the antibacterial agent. Moreover, MPNP is eco-friendly (a natural by-product of citrus fruit) and more economic as it could be recovered and reused. The procedure was evaluated according to the greenness assessment tools: AGREE calculator and Hexagon-CALIFICAMET, showing good green scores, ensuring the process’s eco-friendliness.

**Supplementary Information:**

The online version contains supplementary material available at 10.1186/s13065-023-01048-4.

## Introduction

According to WHO, the number of COVID-19 confirmed cases exceeded 400 million cases by the beginning of the year 2022 [1]. Several pharmaceuticals including antibiotics have been repurposed for the management of COVID-19 by either resolving bacterial infections co-existing with COVID-19 or testing their potential antiviral activities [2, 3]. The risk of environmental bioaccumulation of antibiotics may cause bacterial resistance, genotoxic effects, and endocrine disturbance [4]. Consequently, antibiotics needed to be treated and removed from the ecosystem. Among the most efficient techniques for wastewater treatment is the adsorption technique; it has the advantages of design simplicity, ease of operation, insensitivity to toxic pollutants, and economic when using low-cost adsorbent [5–7].

Fluoroquinolones have been used as adjuncts in treating patients presenting COVID-19, due to their potential antiviral, along with their immunomodulatory properties, favorable pharmacokinetics, and excellent safety profile [8]. Levofloxacin (LEVO), which belongs to this group, is a fluorinated carboxyquinolone [9]. The chemical formula is shown in Additional file 1: Fig. S1. The literature review revealed two published works that used the adsorption technique for the removal of LEVO from water samples. The first one used activated carbon, barley husks, and eggshells with only 74% removal [10], and the second one studied the adsorption of LEVO and phosphate on the goethite (α-FeOOH) surface [11].

Nanomaterials (NM) has attracted the focus as adsorbent; as it has many advantages in water purification such as inertness, high specific surface areas, fast dispersion, high reactivity, and sorption capacity. The safety of the NM is high being less powerful oxidants relative to chemical disinfectants, therefore, the production of harmful by-products is unlikely to happen [12]. The efficiency of NM depends on the structural properties of the material, the adsorbate nature, and conditions of water. The efficiency and specificity of NM can be varied by surface modifications with different inorganic (as silica) or organic/polymeric (as pectin) coating agents [13].

Our study involves the use of adsorptive materials, which are magnetite pectin nanoparticles (MPNP) and mesoporous silica nanoparticles (MSNP). The pectin was considered a low-cost, non-toxic, and, readily available natural by-product from citrus fruit (agricultural waste), and it was used as an adsorbent to remove several organic compounds and metals from water such as methylene blue dye [14], crystal violet dye [15], amoxicillin [16], heavy metals [17] and it was also used in water desalination [18]. There has been a growing interest in the use of silica or mesoporous silica as adsorbent due to its large surface area, large pore volume, availability, and mechanical stability [19]. It was previously used for adsorption of polyvinylpyrrolidone [20], dyes [21–23], organic pollutants [24], and metal ions in water [25]. The main drawbacks of mesoporous silica are its synthesis which needs accurate tuning of many parameters affecting the final structure, and its high cost due to the usage of surfactants and copolymers which limits its use as an adsorbent [25].

The aim of this study is the synthesis of magnetite pectin nanoparticles (MPNP) and their characterization. A comparative adsorption study is carried out against mesoporous silica nanoparticles (MSNP) for removing LEVO residues from wastewater. A full factorial design was used to optimize the adsorption conditions and undergo the comparison between the two types of nano-adsorbents. The whole procedure was assessed by two greenness tools: AGREE and Hexagon, which proved its efficiency and good impact on the environment.

## Material and methods

### Instruments and software

UV–visible 1800 spectrophotometer connected to UV-Probe 2.32 software (Shimadzu, Japan). HPLC Agilent 1200 series, with multiple wavelength detector and micro vacuum degasser with ChemStation software (Agilent Technologies, Germany). Phenomenex Gemini^®^ C_18_ column (150 mm × 4.6 mm, 5µm particle size S/N: H16-292954 from (Agilent Technologies, Polo Alto, CA, USA). Magnetic stirrer (Stuart, England). pH-meter (Jenway3505, UK). Design Expert Software version 7.0 (Stat-Ease Inc., Statistics made easy, Minneapolis, USA). Characterization of the particles was done using Transmission Electron Microscopy (HR-TEM, JOEL JEM-2010), Zeta sizer (Malvern ZS nano), Fourier-transforms Infrared spectrophotometer (FT-IR, JASCO spectrometer), and X-ray diffractometer (Shimadzu XRD 6000 diffractometer).

### Materials and reagents

Levofloxacin (LEVO) was kindly supplied from Sanofi Company, Egypt. The purity was tested by the official USP method [26] and was found to be (99.54 ± 0.67). Pectin (Alfaster), Ferrous chloride (FeCl_2_), and ferric chloride (FeCl_3_) were supplied from (Fisher Scientific, USA). Magnetite silica nanoparticles (MSNP) were purchased from nanotech, Egypt. HPLC grade methanol and acetonitrile were purchased from (Riedel–de Haen, Sigma-Aldrich, Germany). HCl and NaOH were obtained from ADWIC Company, Egypt. Distilled water was used throughout the work.

### Preparations

#### Standard solutions

A stock solution of standard LEVO of concentration (500 µg/mL) was prepared using distilled water. Working standard solutions were prepared by accurately transferring aliquots from the stock solutions to prepare concentrations of 10 μg/mL or 20 μg/mL. The prepared solutions were protected from light by wrapping the flask with aluminum foil and kept at room temperature**.**

#### Synthesis of bare magnetite ferric oxide nanoparticles

The magnetite nanoparticles were synthesized using the co-precipitation method. A mixture of ferric and ferrous ions of ratio (2:1) was dissolved in deionized water under nitrogen gas to avoid the oxidation of the ferric ions to ferrous. A base was fed to the mixture drop-wise at a constant rate under vigorous stirring till the solution turned black indicating the formation of the magnetite nanoparticles. The particles were retrieved by a magnet, washed with distilled water, and dried overnight in the oven at 90 °C [27].

#### Synthesis of magnetite pectin nanoparticles (MPNP)

The pectin was added dropwise to the previously described magnetite ferric oxide nanoparticles under stirring as a final step after the precipitation of the ferric-oxide nanoparticles by the base. The mixture was mixed for two hours at 80 °C. The particles were retrieved by a magnet, washed with distilled water, and dried overnight in the oven at 90 °C. [27, 28].

### Analytical techniques

#### Spectrophotometric analysis

The calibration curve for LEVO was built by plotting the concentrations of its standard solutions prepared in distilled water in the range of (2.5–12 μg/mL) against their corresponding absorbance at λ_max_ (294 nm) [29].

#### Chromatographic conditions

As per our previous study [30], the samples were analyzed using Gemini^®^ C_18_ column (150 mm × 4.6 mm, 5 μm particle size i.d.) and a mobile phase consisting of methanol: 0.05 M phosphate buffer (pH 6) in a ratio 50:50 v/v. The pH of the buffer was adjusted using orthophosphoric acid. The flow rate was 1.5 mL/min and the UV detection at 294 nm. The calibration curve was linear in the concentration range (10–100 μg/mL). The validation parameters were calculated according to the ICH guidelines [31].

### Preliminary studies

Preliminary studies were carried out using spectrophotometric analysis, to determine the factors affecting the adsorption process and the values of these factors to build up the experimental design. The procedure was carried out as follows: 25 mL of either 5, 10 or 20 μg/mL of LEVO working solutions were transferred into a 50 mL beaker, and 4, 8, or 16 g/L of either MPNP or MSNP was added. The pH was adjusted to 4, 5, 6, or 7 using 0.1N HCl or 0.1N NaOH, then the solution was gently mixed for 1 h and left for 4, 8, or 12 h at room temperature. After the contact time, the loaded solutes were separated by an external magnet, the supernatant solution was filtered, and the absorbance was measured at λ_max_ (294 nm).

### Factorial design

The full fractional factorial design (2^4^) was used to study the effect of four factors: the pH, the contact time, the initial concentration of the drug (LEVO), the concentration of the absorbent for two types of adsorbents: MNPN and MSNP. Two levels for each factor were chosen: low level (−1) and high level (+ 1), as shown in Table 1. Thirty-two samples were prepared with different levels of the factors to choose the optimum conditions for the highest adsorption of LEVO, as shown in Table 2. The pH was adjusted to 4 or 7 by adding suitable amounts of 0.1M HCl or 0.1M NaOH solutions. The contact time was set to 4 or 12 h. The initial concentration of LEVO was 10 or 20 μg/mL. The type of adsorbent was MPNP or MSNP with concentrations of 4 or 16 g/L.

**Table 1:** The factors and their levels used for the fractional factorial design (2^4^) experiment

Factor name	Low level (−1)	High level (1)
pH	4	7
Contact time	4 h	12 h
Initial drug Conc	10 µg/mL	20 µg/mL
Concentration of the adsorbent	4 g/L	16 g/L

**Table 2:** Design matrix for the factorial (2^4^) employed for LEVO removal and results of the RP-HPLC using the two adsorbents: MPNP & MSNP

Run no	pH	Time (h)	Levo conc (µg/mL)	Adsorbent conc (g/L)	Average peak area	% Adsorption	Run no.	pH	Time (h)	Levo conc (µg/mL)	Adsorbent conc (g/L)	Average peak area	% Adsorption
	Adsorbent type: MPNP							Adsorbent type: MSNP					
*1*	*4*	*4*	*10*	*16*	*60.99*	*96.55*	17	7	12	20	16	25.04	98.58
2	4	4	20	16	180.45	95.6	*18*	*7*	*12*	*10*	*16*	*45.44*	*98.89*
3	7	4	10	16	681.14	61.42	19	7	4	10	16	39.36	97.77
4	4	4	10	4	123.25	93.02	20	4	12	10	16	31.65	98.21
5	7	12	20	4	1927.52	52.98	21	4	4	20	4	215.67	94.74
6	4	12	20	16	181.3	95.58	22	7	4	20	16	71.11	98.27
7	4	12	10	16	140.3	96.52	23	7	4	20	4	215.35	94.75
8	4	12	20	4	1502.17	63.35	24	4	12	10	4	100.1	94.33
9	4	4	20	4	1348.77	67.1	25	7	4	10	4	41.66	97.64
10	7	12	10	4	964.04	45.4	26	4	12	20	16	75.54	98.16
11	7	12	10	16	1030.52	41.63	27	4	12	20	4	135.46	96.7
12	7	12	20	16	1558.39	61.98	28	4	4	10	4	214.1	87.87
13	7	4	20	16	2646.19	35.44	29	4	4	10	16	35.99	97.96
14	7	4	10	4	660.17	62.61	30	7	12	10	4	45.33	97.43
15	4	12	10	4	437.85	75.2	31	4	4	20	16	90.6	97.79
16	7	4	20	4	544.12	66.48	32	7	12	20	4	137.3	96.65

After the contact time, the loaded adsorbent was collected by a magnet and the supernatant samples were filtered using syringe filter paper (0.45 μm, PTFE) and injected into the chromatographic system to determine the amount of LEVO left in the supernatant solutions after the specific contact time. The adsorption percentage was calculated as follows:

$${{\% \,{\text{adsorption}} = \left( {{\text{C}}^{\prime} - {\text{C}}} \right)} \mathord{\left/ {\vphantom {{\% \,{\text{adsorption}} = \left( {{\text{C}} - {\text{C}}} \right)} {{\text{C}} \times 100}}} \right.} {{\text{C}}^{\prime} \times 100}}$$

where Cʹ is the concentration of the initial drug solution and C is the concentration of the treated drug solution.

### Calculation of adsorption isotherms and models

A fixed concentration of MPNP of 0.1 g/L was added to a range of concentrations of LEVO concentrations (1–25 g/L). The volume of all the samples was kept constant at 10 mL and the volume was completed with distilled water. The pH was adjusted to 4 with 0.1N HCl. The mixtures were shaken gently for 1 h and left to equilibrate for four hours. The magnetite nanoparticles were then collected by a magnet and the remaining solution was filtered through a 0.25 µm Millipore syringe filter. The filter solution was then measured spectrophotometrically at LEVO λ_max_ 294 nm.

At equilibrium, the adsorbed amount of LEVO q_e_ (mg/g) was calculated using the following equation, referred to as the mass balance equation which is expressed as:

1 $$q_{e} = { }\frac{{v{ }\left( {C_{i} - C_{e} } \right)}}{m}$$

Where C_i_ and C_e_ (mg/L) are the initial and equilibrium concentrations of LEVO, respectively, v (L) is the total volume of the samples, and m (g) is the mass of the dry MPNP [1]. The equilibrium data were analyzed using four isotherms: Langmuir, Freundlich, Redlich-Peterson, and Sips.

## Results and discussion

### Characterization of magnetite pectin nanoparticles

The proposed magnetite pectin nanoparticles (MPNP) were prepared using co-precipitation method. This synthesis method was selected to be green, facile and one-pot-method if compared with other methods. The MPNP could be recovered from the water samples prior to treatment by imparting magnetite property to the pectin which plays an important role for economic regeneration of the adsorbent. The magnetite property was imparted by using ferric oxide nanoparticles, prepared by coprecipitation method which is coated with pectin afterwards through a (COO–Fe) linkage [32–34]. The particles were characterized using the following methods:

#### Transmission electron microscope (TEM***)***

**Fig. 1:**
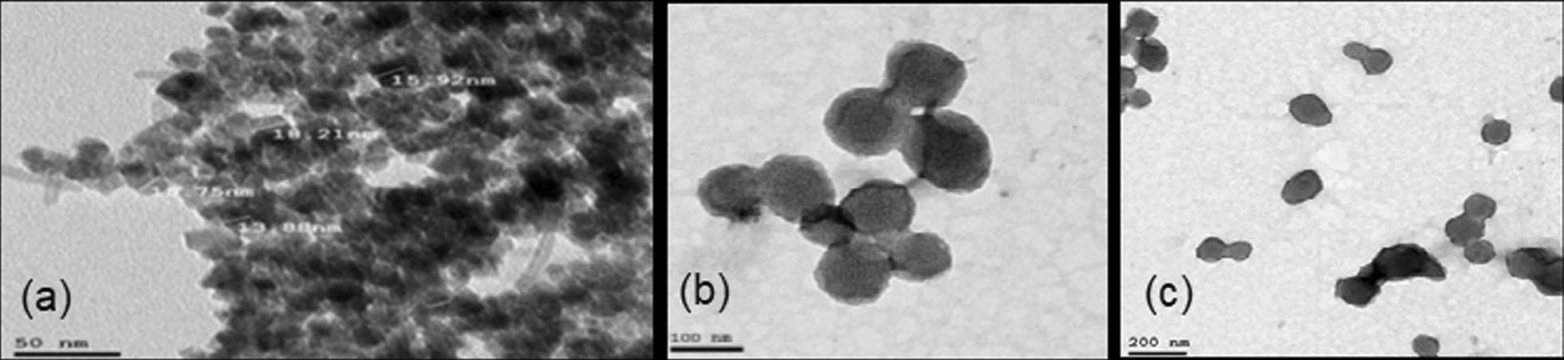
TEM images of **a** MPNP with core average size of approximately; MSNP at different scale **b** 100 nm and **c** 200 nm

TEM delivers direct images, from which information on size and shape of nanoparticles is obtained. It was used to determine the size of the core and shell of the particles due to the good contrast provided by the different nature of the ferric-oxide-based core (which has darker color) and the polymer-based shell (lighter color). The smaller the particles, the higher is the sorption capacity of antibiotic to the magnetic nanoparticles (MNPs) and the magnetic capacity [13]. Therefore, tuning the size of the particles to small size 15 nm and 90 nm for the MPNP, Fig. 1a, and MSNP, Fig. 1b, c, was necessary to ensure the efficiency of the purification process.

#### Zeta potential

It measures the surface charge of the particles and determines the stability of the colloidal dispersion. Several works report that the removal efficiency is influenced by the adsorbent surface charge, hydrophobicity and surface properties and the adsorbent [35]. For MPNP, the values for bare (uncoated) Fe_3_O_4_ nanoparticles were – 17 mV which indicates that the colloids are negatively charged but the colloidal dispersion is unstable. On the other hand, the coated Fe_3_O_4_ showed a value of – 27 mV reflecting the negative charge imparted by the pectin coated and the enhanced stability of the colloidal dispersion relative to bare particles. For comparison purpose the Zp values of the mesoporous silica was measured to be − 17.7 mV in agreement with the values reported in literature [36, 37].

#### Fourier transforms infrared spectroscopy (FT-IR)

**Fig. 2:**
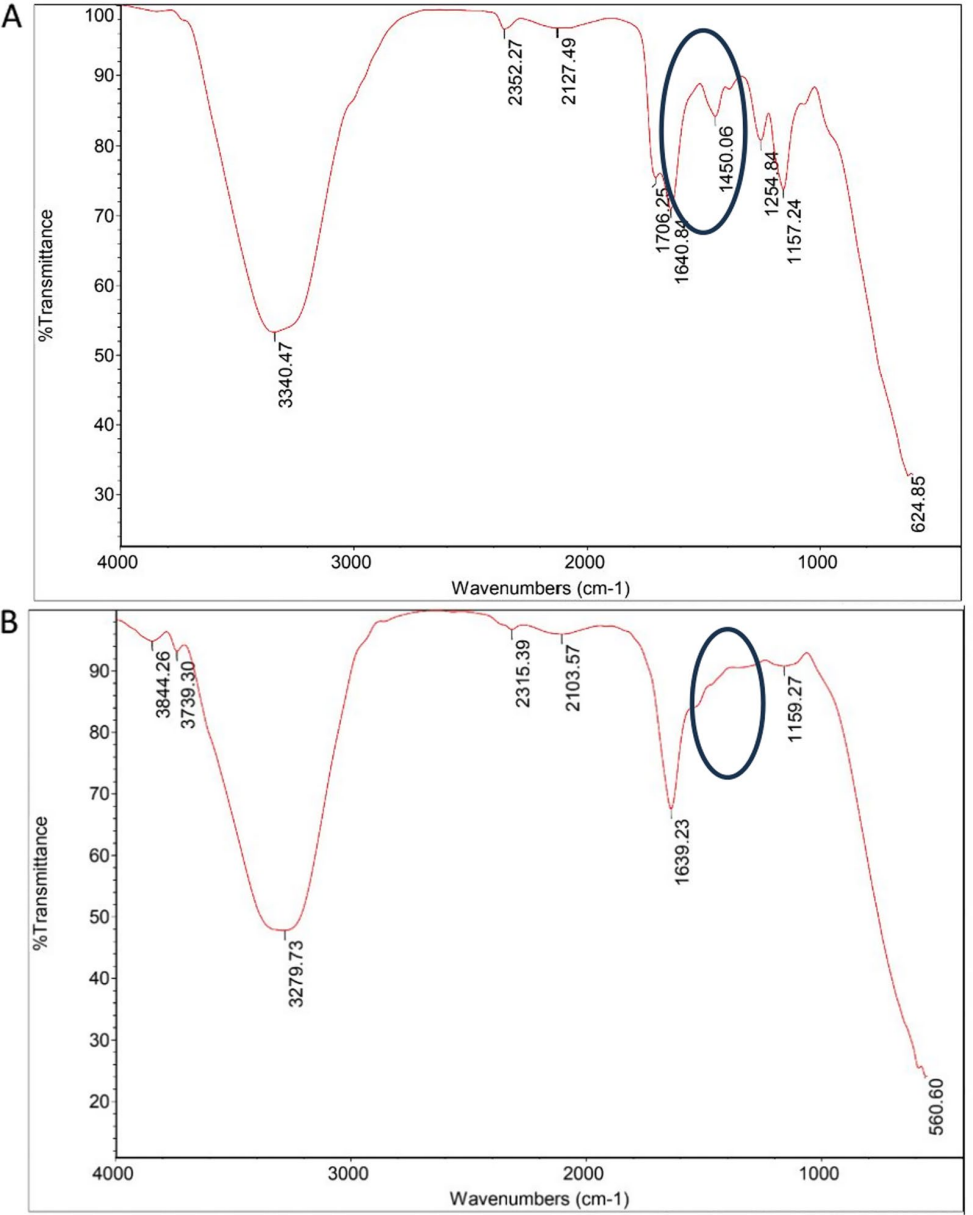
IR spectrum of **A** MPNP nanoparticles and **B** bare Fe_3_O_4_ nanoparticles

The IR spectrum illustrates that the pectin polymers successfully coated the Fe_3_O_4_ particles via forming a COO–Fe with a characteristic absorption at 1393 cm^1^ and 1587 cm^1^ [38] as shown in Fig. 2

#### X-Ray diffraction (XRD***)***

**Fig.3:**
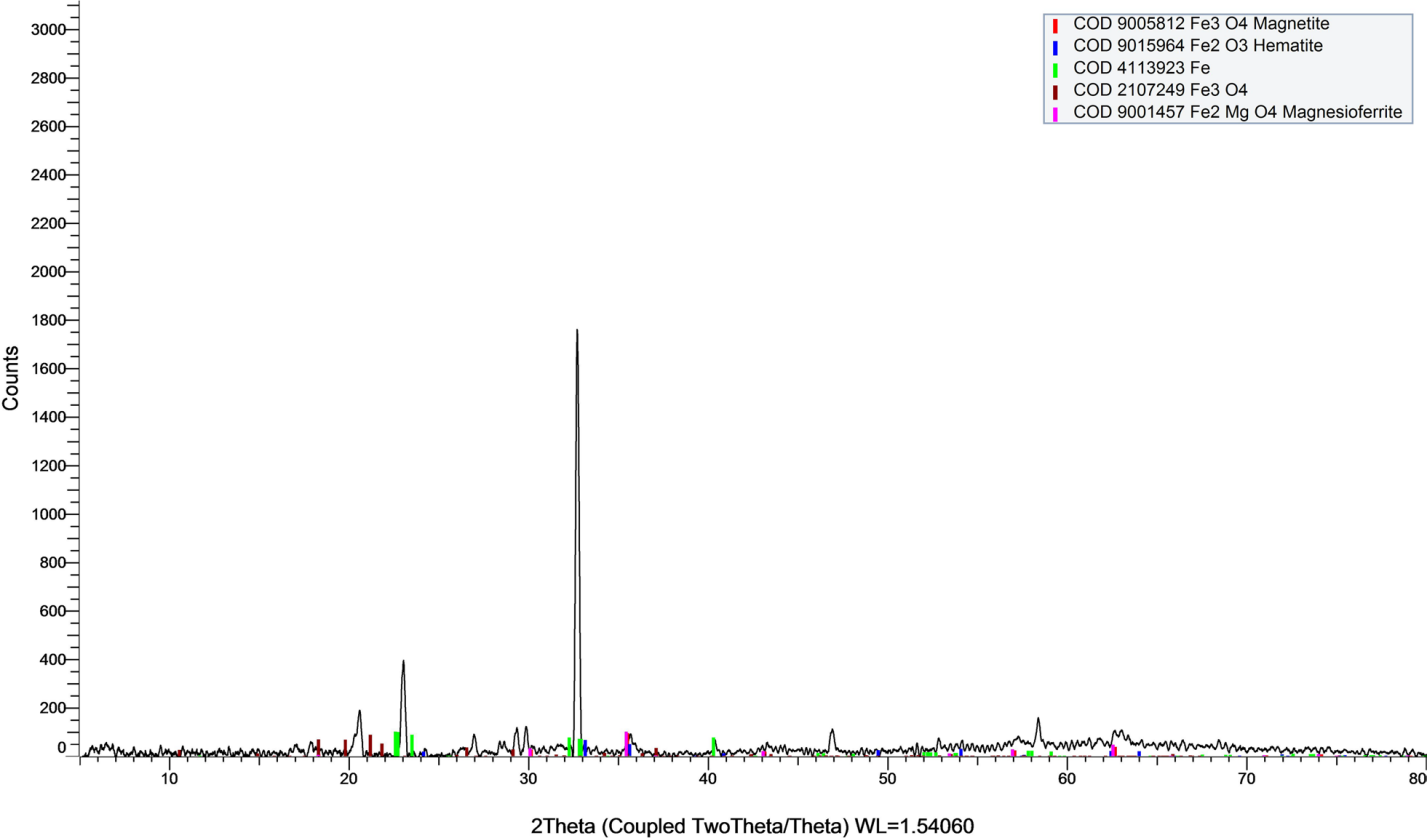
X-Ray diffraction illustrating cubic phase of MPNP particles

It confirmed the crystalline structure of the Fe_3_O_4_ nanoparticles, and it is not changed by coating with the pectin polymers where a cubic phase has been shown in Fig. 3**.**

### Chromatographic assay

**Fig. 4:**
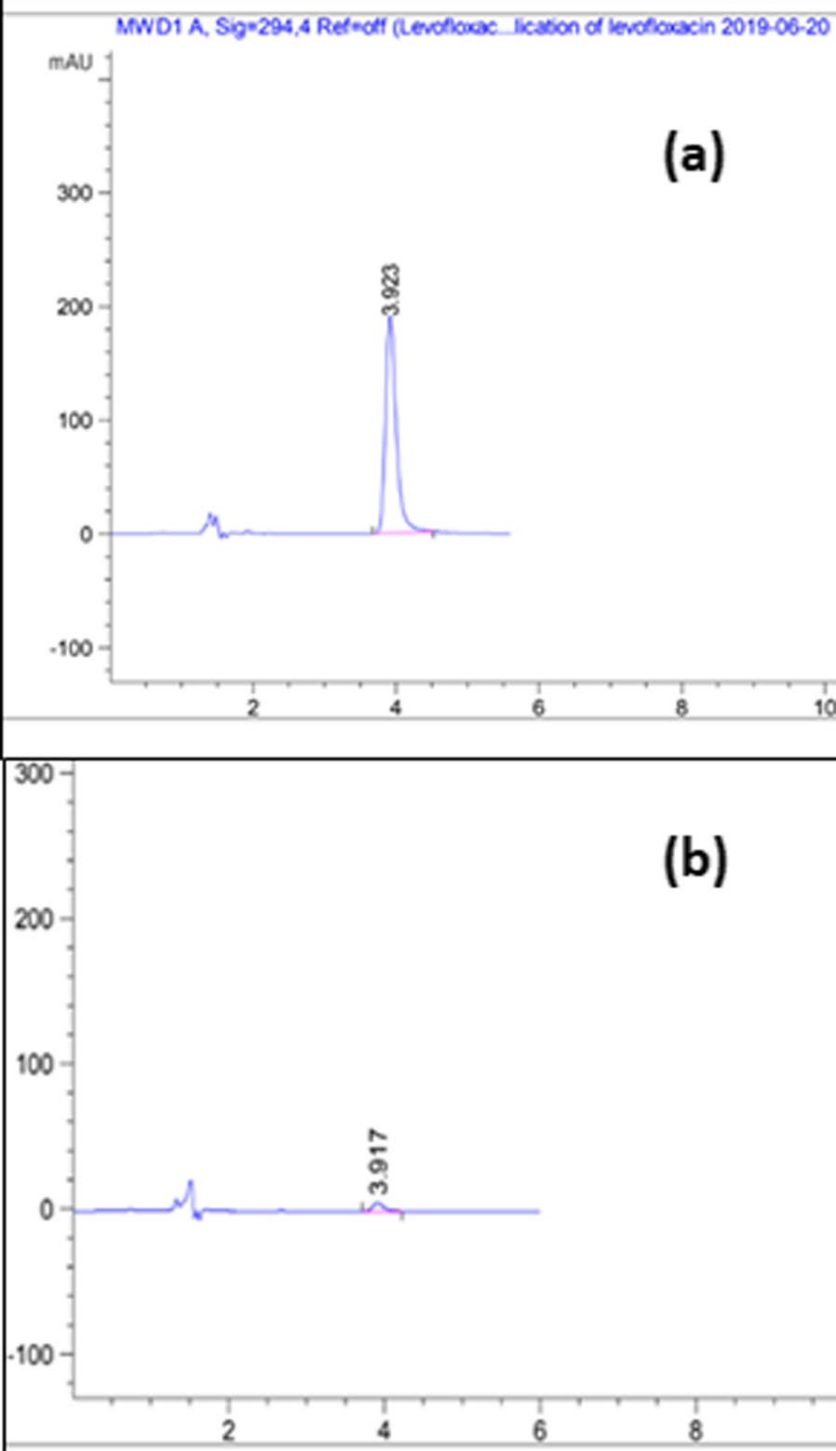
Chromatograms of (**a**) intact LEVO (10 µg/mL), (**b**) LEVO (10 µg/mL) treated with MPNP (16 g/L) at pH 4 and contact time 4 h

In this experiment, isocratic elution was employed at a flow rate of 1.5 mL/min and UV detection at 294 nm. The obtained regression equation (y = 23.784x + 22.649) was used to calculate the concentration of LEVO residual after the adsorption process. The chromatograms of intact LEVO before and after treatment using MPNP and MSNP are shown in Fig. 4. A significant decrease in the peak area of LEVO was observed which proves the adsorption of the drug on the surface of the two nanoparticles. The system suitability and validation parameters for the proposed HPLC/UV method are demonstrated in Table 3.

**Table 3:** System suitability and validation parameters for the proposed HPLC/UV method

Parameters		HPLC	Reference values^*^
Linearity (µg/mL)		10–100	
Correlation coefficient (r)		0.9997	
Slope		23.784	
Intercept		22.649	
Standard deviation of residuals from line		21.325	
LOD (µg/mL)		2.959	
LOQ (µg/mL)		8.966	
Accuracy (Recovery % ± SD)^a^		101.03 ± 0.86	
Precision (RSD)	Intraday^b^	99.36 ± 0.97	
	Interday^c^	100.11 ± 1.33	
t_R_, min		3.916 ± 0.05	
Tailing factor (T)		0.78	T ≤ 2, T = 1 for symmetric peak
Capacity factor (Kʹ)		1.42	Kʹ = 1–10 acceptable
Plates number (N)		6255	N > 2000
Height equivalent to theoretical plate (HETP; cm plate^−1^)		0.03	The smaller the value, the higher the column efficiency

^a^Average of three blind concentrations analyzed in triplicate

^b^Average of three concentrations (25, 50, 75 µg/mL) analyzed in triplicate on the same day

^c^Average of three concentrations (25, 50, 75 µg/mL) analyzed in triplicate on three successive days

* according to USP

### Experimental design

The spectrophotometric preliminary studies gave guidance for choosing the levels of factors to build the design Additional file 1: Fig. S2. The full factorial design (2^4^) was employed to study the significance of each factor (pH, contact time, drug concentration, and concentration of the adsorbent) on the adsorption process, in addition, to select the optimum conditions for maximum adsorption of LEVO by MPNP and MSNP. Thirty-two samples were analyzed under variable conditions by the adopted chromatographic conditions.

The optimum adsorption conditions were selected by scoring the lowest peak area of LEVO after treatment which indicated the best adsorption percentage. These optimum conditions were found to be pH 4 and contact time of 4 h for MPNP; while for the MSNP, the optimum conditions were found to be pH 7 and 12 h of contact time, with drug and nanoparticles concentration of 10 µg/mL and 16 g/L, respectively for both nanoparticles. The results of adsorption efficiency are shown in Table 2**.**

### Evaluation of the adsorption efficiency

#### Effect of pH

Chemical adsorption occurs between the nanoparticles and our studied drug (LEVO), where an electron is transferred, and a chemical bond is formed between the adsorbate (LEVO) and the solid surface of the nanoparticles. Chemical adsorption is stronger and more specific than the physical type of adsorption (which depends on the weak van der Waals forces) [39]. The hydroxyl group on the MPNP surface plays an important role in chemical adsorption. The MPNP surface undergoes a pH-dependent protonation/ deprotonation [40] which takes place as follows [41]:

$${-}{\text{Fe}}{-}{\text{O}}{-}\left( {\text{C O}} \right){-}\left( {{\text{OH}}} \right)_{{\text{n}}} \, + {\text{ H}}^{ + } \leftrightarrow {-}{\text{Fe}}{-}{\text{O}}{-}\left( {\text{C O}} \right){-}\left( {{\text{OH}}_{2} } \right){\text{n}}^{ + }$$

**Fig. 5.**
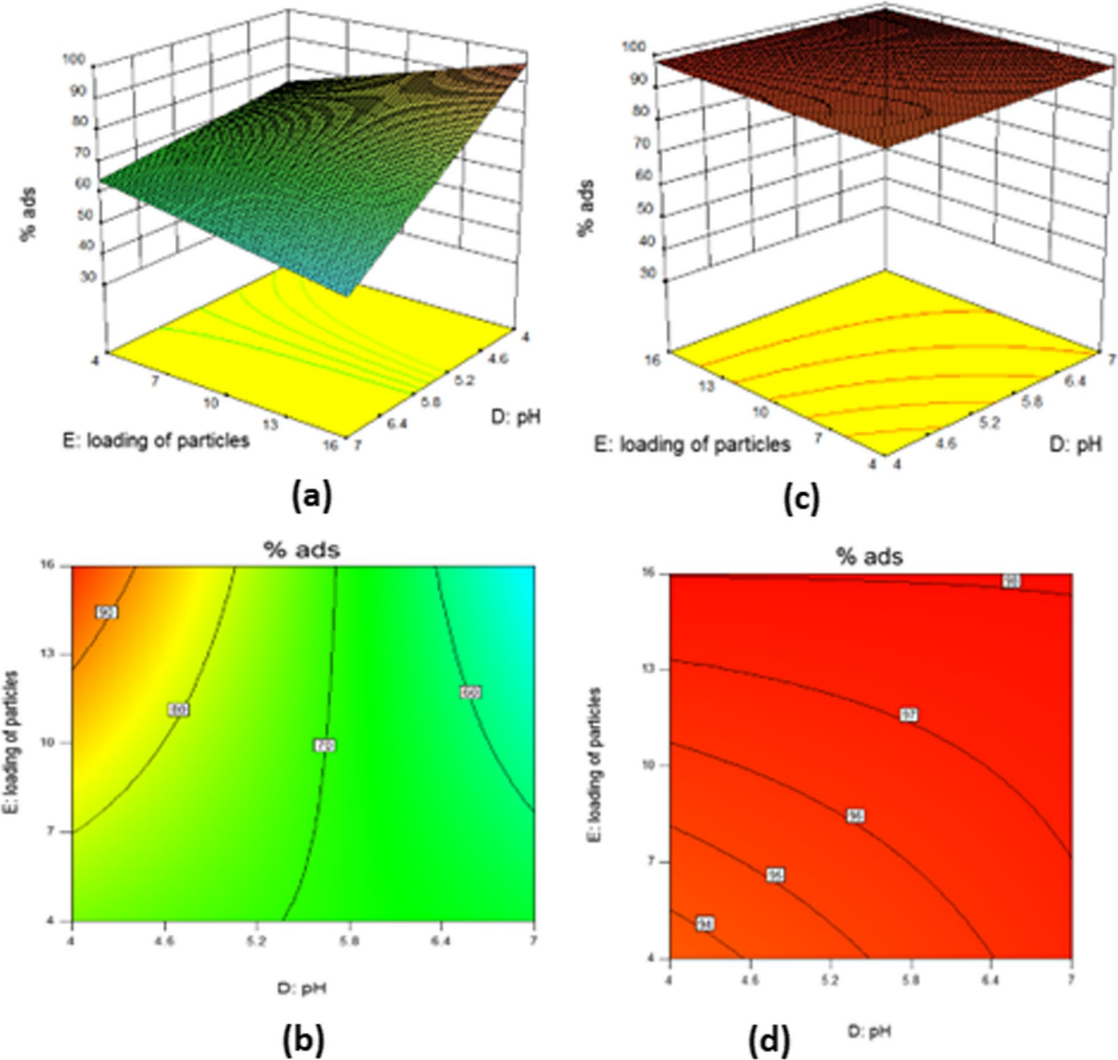
3D plot (**a**) and contour plot (**b**) of adsorption % of LEVO samples by MPNP as a result of effect of pH and nanoparticles concentration and the other factors are kept constant; 3D plot (**c**) and contour plot (**d**) of adsorption % of LEVO samples by MSNP as a result of effect of pH and nanoparticles concentration and the other factors are kept constant

At pH < pH _pic_ of MPNP (which is 5.21), the positive species [Fe–O–(C O)–(OH_2_)n^+^] is the dominant form. On the other hand, LEVO has 2 pK_a_ values (5.59 and 7.94); at pH < 5.59 the carboxylic acid group dissociates carrying a negative charge [42]. Consequently, electrostatic attraction force between MPNP and LEVO molecules was favored at pH 4, and the adsorption was enhanced when compared to the adsorption at pH 7, as shown in the 3D and contour plots, Fig. 5a, b. At pH < 4, the LEVO adsorption was decreased due to the competition between H + and the cationic LEVO for the adsorption sites of MPNP (containing OH^−^ groups).

For the MSNP, at acidic pH (< pH 7), the surface of the silica becomes positively charged which favors the electrostatic attraction with LEVO and thus the adsorption. While at elevated pH, the silanol groups (Si–O) are negatively charged which causes electrostatic repulsion between negatively charged sites on the MSNP surface and cationic LEVO and thus inhibits the adsorption process. The ionization of MSNP takes place according to the following equation:

SiOH_2_^+2^ ↔ SiOH ↔ SiO^−^ + H^+^ [43]

The MSNP acquires a positive charge at the two proposed pH levels (pH 4 and 7). As per the experimental design results, the two pH levels did not have a significant difference in the efficiency of the adsorption process. According to the results of the factorial design, a pH of 5.7 was found to be the optimum value for the adsorption process, as shown in the 3D and contour plots of Fig. 5c, d.

#### Effect of drug and the nanoparticles concentrations

In general, an increase in adsorbate (drug) concentration can lead to lower adsorption efficiency. On the other hand, an increase in adsorbent (nanoparticles) load will result in better adsorption efficiency but the operating cost will increase. The initial concentrations of adsorbate (the drug) and adsorbent (nanoparticles) will determine when the adsorption becomes saturated. The studied levels of drug concentrations were 10 and 20 µg/mL while the concentrations of MPNP and MSNP particles were 4 and 16 g/L. The maximum adsorption percentage of 10 µg/mL LEVO by 96.55% was obtained by using 16 g/L MPNP at pH 4. While the adsorption percentage reaches 98.89% for the same concentration of LEVO by using 16 g/L MSPN at pH 7, as shown in runs no. 1 and 18 in Table 2.

#### Effect of contact time

Contact time is an important factor in designing a wastewater treatment method. It is preferred that the contact time between adsorbate and adsorbent will be as minimum as possible with maximum adsorption. The optimum contact time was obtained by measuring the time needed to reach the equilibrium using a fixed concentration of LEVO and at natural pH. It was found that the optimum contact time for MPNP was 4 h; while for MSNP, maximum adsorption was achieved by 12 h.

The optimum adsorption conditions and desirability indexes are shown in Additional file 1: Fig. S3a for MPNP and Additional file 1: Fig. S3b for MSNP. Both particles showed close desirability indexes and adsorption capacities for LEVO. Moreover, the fabricated MPNP had the advantage of saving the contact time by 8 h when compared to MSNP. The regeneration of MPNP was done using methanol to be reused for further wastewater treatment processes.

### Sorption isotherms

Sorption isotherms illustrate the partitioning of the adsorbate between the liquid phase and MPNP based on the adsorbent heterogeneity or homogeneity assumptions, adsorbate–adsorbate interaction, and coverage type [44]. The required parameters for adsorption isotherms are presented in Table 4. The difference shown along the values of the initial levofloxacin concentration (C_i_) and its equilibrium concentration (C_e_) illustrates the occurrence of the adsorption process, where a decrease in the levofloxacin concentration was observed. In addition, the amount of the adsorbed drug per unit mass of MPNP (q_e_) was calculated and presented in Table 4.

**Table 4:** Parameters required for the kinetics adsorption study

C_i_ (mg/L)	C_e_ (mg/L)	q_e_ (mg/g)
1	0.485436893	5.145631068
2	0.524878641	14.75121359
3	0.773665049	22.26334951
4	0.970873786	30.29126214
5	1.277305825	37.22694175
10	2.545509709	74.54490291

The sorption data were fitted to four adsorption isotherm models: Langmuir, Freundlich, Redlich-Peterson, and Sips isotherms, to determine which isotherm better describes the data based on the quality of fit. All experiments were performed at room temperature being the suitable for larger scale water purification process. A brief introduction for each model is illustrated in the next few paragraphs.

#### Langmuir isotherm

Langmuir adsorption isotherm assures that adsorption energy is constant, with no interaction between adsorbate molecules. The saturation of the adsorbate surface occurs by the formation of a monolayer of the adsorbent which indicates maximum adsorption. Langmuir isotherm linear expression can be represented by:

2 $$\frac{1}{{q}_{e}}=\frac{1}{{q}_{m }{K}_{l}} \frac{1}{{C}_{e}}+ \frac{1}{{q}_{m}}$$

A linear graph is obtained by plotting 1/q_e_ *versus* 1/C_e_. The intercept and slope of the linear plot express K_L_ (L/mg) as the Langmuir energy constant whereas Q_m_ (mg/g) is the maximum amount of antibiotic adsorbed per unit mass of MPNP. The R_L_ is a dimensionless constant factor that could be calculated from the Langmuir isotherm, which expresses either the adsorption is favorable or not.

3 $${R}_{L}= \frac{1}{( 1+ {K}_{L}{C}_{i})}$$

where Ci is the highest adsorbate concentration [45].

The R_L_ value is between 0 and 1 the adsorption is favorable. From the values presented in Table 5, the R_L_ value (0.94) indicates that the adsorption is favorable, and the regression coefficient, R^2^ = 0.9446 indicates that this model is describing the data set well as in Additional file 1: Fig. S4.

**Table 5:** Parameters and correlation coefficients (R^2^) of the isotherm models for the data of Levofloxacin adsorption to MPNP

Langmuir		Frendlich		Reddlich-Peterson		Sips	
K_L_	0.006 (L/mg)	K_F_	1.4054 (1/g)	K_R_	2.755 (mg/g)	K_s_	0.0023 (L/mg)
q_m_	0.0307 (mg/g)	1/n	− 1.359	a_R_	3.23 (mg/L)	Q_max_	0.0837 (mg/g)
R_L_	0.94			b_R_	0.359		
R^2^	0.9446	R^2^	0.8495	R^2^	0.8522	R^2^	0.9916

#### Freundlich isotherm

Freundlich isotherm assumes that the adsorption sites have various adsorption energies as well as multilayer adsorption is presumed. The linear expression of the isotherm can be expressed by the following:

4 $$og{q}_{e}= \frac{1}{n}\mathrm{log}{C}_{e}+\mathrm{log}{K}_{F}$$

The linear graph can be obtained by plotting log C_e_ *versus* q_e_. The Freundlich isotherm parameters K_F_ (1/g) and 1/n can be obtained from the intercept and slope, respectively. The K_F_ represents the Freundlich adsorption capacity constant and 1/n the intensity constant of the adsorption [46].

The Freundlich isotherm parameters, as well as the correlation coefficients (R^2^ = 0.85), are listed in Table 5, which indicates that this model doesn’t best describes the data sets as shown in Additional file 1: Fig. S5.

#### Redlich-Peterson isotherm

It is a three-parameter adsorption model that doesn’t assume ideal monolayer adsorption. It is a combination of elements from Langmuir and Freundlich isotherms. The linear equation of the model can be given by:

5 $$m\left({K}_{R}\frac{{C}_{e}}{{q}_{e}}-1\right) {b}_{R}\mathrm{ln}{C}_{e}+\mathrm{ln}{a}_{R}$$

K_R_ is the Redlich-Peterson adsorption capacity constant, a_R_ is isotherm constant, and b_R_ is the exponent. Langmuir and Freundlich isotherms can be obtained from Redlich-Peterson isotherm. If the b_R_ value equals to 1 then the equation can be reduced to Langmuir and a value of zero reduces the equation to Freundlich [47].

The Redlich-Peterson isotherm parameters as well as the correlation coefficients (R^2^ = 0.85) are presented in Table 5, which indicates that this model doesn’t best describes the data sets as shown in Additional file 1: Fig. S6.

#### Sips isotherm

Like Redlich-Peterson isotherm, it is a three-parameter model assumes a localized adsorption with adsorbate–adsorbate interaction. It is also derived from Langmuir and Freundlich isotherms. Sips linear model can be expressed by:

6 $$\frac{1}{{q_{e} }} = { }\frac{1}{{Q_{max} K_{s} }}{ }\left( {\frac{1}{{C_{e} }}} \right)^{{{\raise0.7ex\hbox{$1$} \!\mathord{\left/ {\vphantom {1 n}}\right.\kern-0pt} \!\lower0.7ex\hbox{$n$}}}} + { }\frac{1}{{Q_{max} }}$$

Where K_s_ (1/mg) is Sips equilibrium constant, Q_max_ (mg/g) is the maximum adsorption capacity, and n is a heterogeneity factor. The n value is bsetween 0 and 1. If n-0, Freundlich equation is obtained whereas a value of 1 gives Langmuir equation [44].

The Sips isotherm parameters are presented in Table 5 and the data fitting is shown in Additional file 1: Fig. S7. Since the utilization of a three-parameter isotherms such as the Sips isotherm model better describe the data sets indicated by the highest R^2^ value (0.9916) obtained from the fit. It may be concluded that the sorption of levofloxacin is attributed to multisite interactions. These findings agree well with Humelnicu et al*.* where the arsenic contaminants adsorption on Amidoxime Resin Hosted by Mesoporous Silica followed sips isotherm where multiple adsorption sites were concluded [48].

### Greenness assessment

#### AGREE calculator

The Analytical greenness calculator is a detailed tool used for the evaluation of analytical procedures according to the 12 principles of green analytical chemistry, where each criterion is assessed and transformed into a scale ranging from 0 to 1. Finally, these scores are collected and presented as a pictogram indicating the final score which reflects the performance of the procedure and showing representative color for each criterion. The greenest procedure shall score unity, while the least green will approach zero [49, 50].

**Fig. 6:**
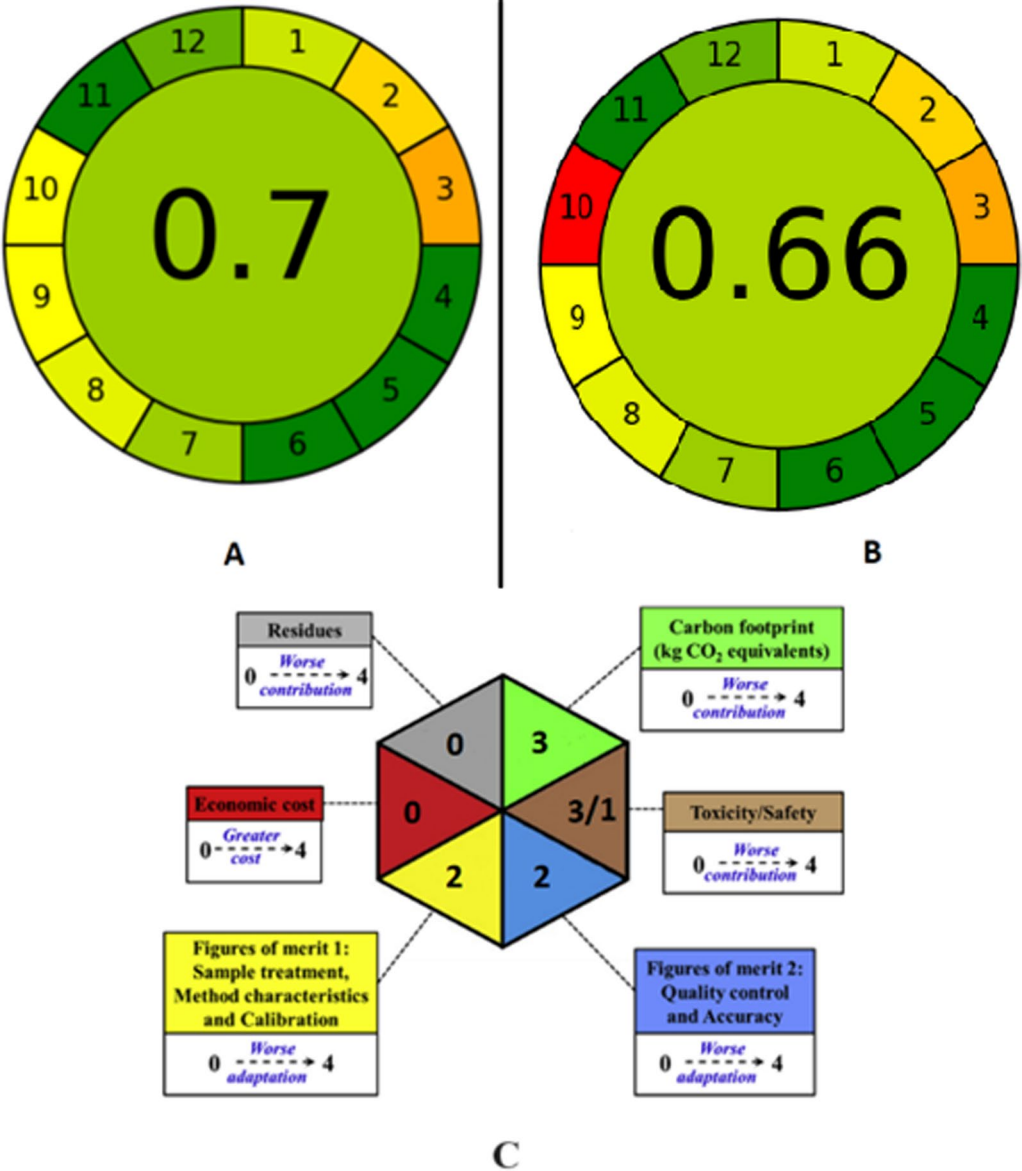
**a** AGREE pictogram of MPNP, **b** AGREE pictogram of MSNP, **c** Hexagon pictogram for the evaluation of the greenness of the proposed MPNP

By studying the performance of the water treatment using MPNP and MSNP, both particles showed a total AGREE score of 0.70 and 0.66, respectively. This reflects a good greenness profile for both particles. Several criteria showed a full score of 1.0 including the integration and ease of operation, reduced used of solvents, saving energy, avoiding derivatization, high level of automation and miniaturization, reducing toxic reagents and high operator’s safety. Minimal waste production is achieved by using both particles due to the economic regeneration of the adsorbent from water samples due to its magnetite property. Both procedures showed lower scores for a few criteria such as at-line analysis of samples and minimum amounts of analytes treated (LEVO only). Ultimately, MPNP particles showed a higher score in criterion no. 10, due to the manufacture of pectin from natural renewable resources (such as citrus peel). The AGREE pictogram is shown in Fig. 6a and b**,** and the detailed AGREE reports are listed as Additional file 1: Table S1.

#### Hexagon-CALIFICAMET

The hexagon-CALIFICAMET tool consists of six equilateral triangles which evaluates six categories of parameters according to penalty points (Pps). As the penalty points increase, the worse the adaptation of the assigned category [51]. The quality parameters are divided into two groups; the first is figures of merit 1 (FM-1) which calculates Pps of sample treatment, method characteristics and calibration as listed in Additional file 1: Table S2. The second group is figures of merit 2 (FM-2) which calculates Pps of quality control and accuracy as listed in Additional file 1: Table S3 The evaluation of toxicity and safety parameters relied on calculating Pps according to the pictograms present in globally harmonized system (SGA) of the reagents used, as listed in Additional file 1: Table S3. Pps are calculated depending on the amount of produced waste and the estimated annual economic cost of the procedure, as listed in Additional file 1: Table S4.

Finally, the Pps for each parameter were calculated and then transformed to a five-level scale ranging from 0 (best) to 4 (worst). The overall qualification was performed for MPNP particles due to its higher AGREE score, listed in Additional file 1: Table S5. It represented each of the previous parameters in an equilateral triangle, in addition to the environmental impact which is expressed by kilograms of CO_2_ equivalent which is known as “carbon footprint”. The hexagon pictogram shown in Fig. 6c shows a score of 0 for waste and cost, due to the regeneration of the adsorbent from water samples and reusing it, in addition to the use of simple and inexpensive apparatus. Safety including physical hazards showed a low score of 1 which indicates the safe reagents used in the procedure. Figures of merit (FM-1) and (FM-2) showed a low score of 2 which indicates good quality of analytical method. Health and environmental hazards showed a moderate score of 3 which indicated mild toxicity of the used reagents. Carbon footprint showed a moderate score of 3 which indicated mild electricity consumption by equipment per analysis time.

## Conclusion

In this work, MPNP were synthesized and characterized. A comparison study was conducted between the fabricated MPNP and an established model of adsorbent (MSNP) for the adsorption efficiency of LEVO from simulated wastewater. The full factorial design was used to reach the optimum conditions for maximum adsorption for both types of adsorbents (MPNP and MSNP). The analysis was done using a validated chromatographic procedure. It was found that MPNP were as efficient as MSNP for removing the antibacterial agent. In addition, our synthesized MPNP have advantages over the MSNP; having simpler method of preparation, saving contact time, being eco-friendly (natural by-product from citrus fruit) and more economic as it could be recovered and reused for successive water treatment. In conclusion, MPNP are promising alternatives for antibiotics removal from wastewater which represents a threat to the environment and human health. The whole procedure was assessed by two greenness tools: AGREE and Hexagon, which proved its efficiency and good impact on the environment. We believed that this approach could be applied for adsorption of further antibiotics from wastewater samples.

### Supplementary Information

**Additional file 1: Fig. S1.** Chemical structure of Levofloxacin (LEVO). **Fig. S2.** The absorption spectra of 20 µg/mL intact LEVO (**___**) and LEVO treated with MPNP (16 g/L) (….) at pH 4 for 4 hrs contact time. **Fig. S3.** The optimum conditions (showing the factors values for pH, initial drug concentration, contact time and the MSNP concentration) for the maximum adsorption of LEVO by **a** MPNP, **b** MSNP. **Fig. S4.** Langmuir adsorption isotherm and data of Levofloxacin adsorption to MPNP. **Fig. S5.** Frendlich adsorption isotherm and data of Levofloxacin adsorption to MPNP. **Fig. S6.** Redlich-Peterson adsorption isotherm and data of Levofloxacin adsorption to MPNP. **Fig. S7.** Sips adsorption isotherm and data of Levofloxacin adsorption to MPNP. **Table S1.** AGREE reports of the proposed procedures. **Table S2.** Figures of Merit 1 (FM-1) of Hexagon-CALIFICAMET. **Table S3.** Figures of Merit 2 (FM-2), toxicity and safety of Hexagon-CALIFICAMET. **Table S4.** Penalty points (PPs) to assess waste generation and annual economic cost of Hexagon-CALIFICAMET. **Table S5.** Overall qualification (OQ) of the variables of the method according to penalty points ranges.

## Data Availability

All data generated or analysed during this study are included in this published article and its Additional files.
