# Supplementary material for: Green wastewater treatment of repurposed COVID-19 therapy (levofloxacin) using synthesized magnetite pectin nanoparticles, comparison with mesoporous silica nanoparticles
Source: BMC Chem. 2023 Oct 9;17(1):134. doi: 10.1186/s13065-023-01048-4 (PMC10563343; doi:10.1186/s13065-023-01048-4)
Supplement: Supplementary file 1 — Additional file 1: Fig. S1. Chemical structure of Levofloxacin (LEVO). Fig. S2. The absorption spectra of 20 µg/mL intact LEVO (___) and LEVO treated with MPNP (16 g/L) (….) at pH 4 for 4 hrs contact time. Fig. S3. The optimum conditions (showing the factors values for pH, initial drug concentration, contact time and the MSNP concentration) for the maximum adsorption of LEVO by a MPNP, b MSNP. Fig. S4. Langmuir adsorption isotherm and data of Levofloxacin adsorption to MPNP. Fig. S5. Frendlich adsorption isotherm and data of Levofloxacin adsorption to MPNP. Fig. S6. Redlich-Peterson adsorption isotherm and data of Levofloxacin adsorption to MPNP. Fig. S7. Sips adsorption isotherm and data of Levofloxacin adsorption to MPNP. Table S1. AGREE reports of the proposed procedures. Table S2. Figures of Merit 1 (FM-1) of Hexagon-CALIFICAMET. Table S3. Figures of Merit 2 (FM-2), toxicity and safety of Hexagon-CALIFICAMET. Table S4. Penalty points (PPs) to assess waste generation and annual economic cost of Hexagon-CALIFICAMET. Table S5. Overall qualification (OQ) of the variables of the method according to penalty points ranges. [file 13065_2023_1048_MOESM1_ESM.docx]

**Green Wastewater Treatment of Repurposed COVID-19 Therapy (levofloxacin) using Synthesized Magnetite Pectin Nanoparticles, Comparison with Mesoporous Silica Nanoparticles**

**Christine M. El-Maraghy^1^, Sarah S. Saleh^*1^, Mervat S. Ibrahim^2^, Omnia A. El-Naem^1^**


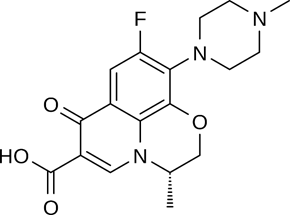


Fig. S1. Chemical structure of Levofloxacin (LEVO)

Fig. S2. The absorption spectra of 20 µg/mL intact LEVO (**___**) and LEVO treated with MPNP (16 g/L) (….) at pH 4 for 4 hrs contact time


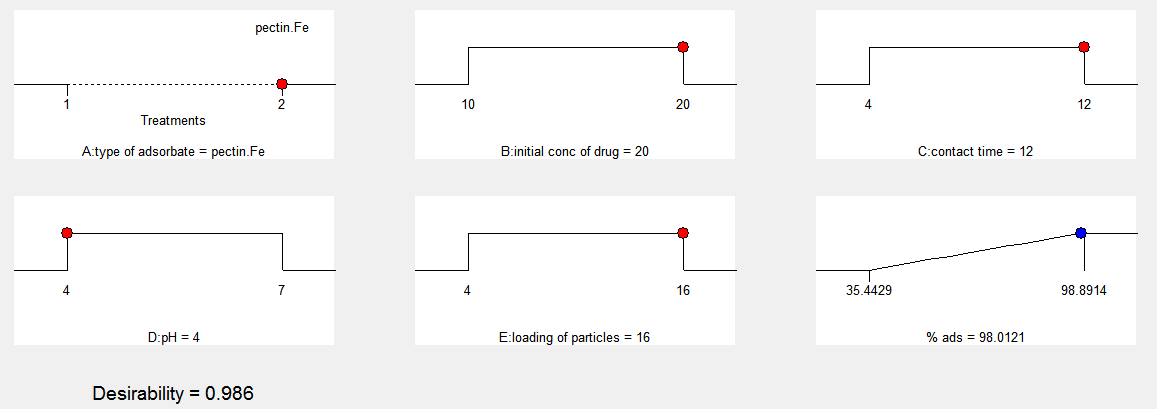


**a**


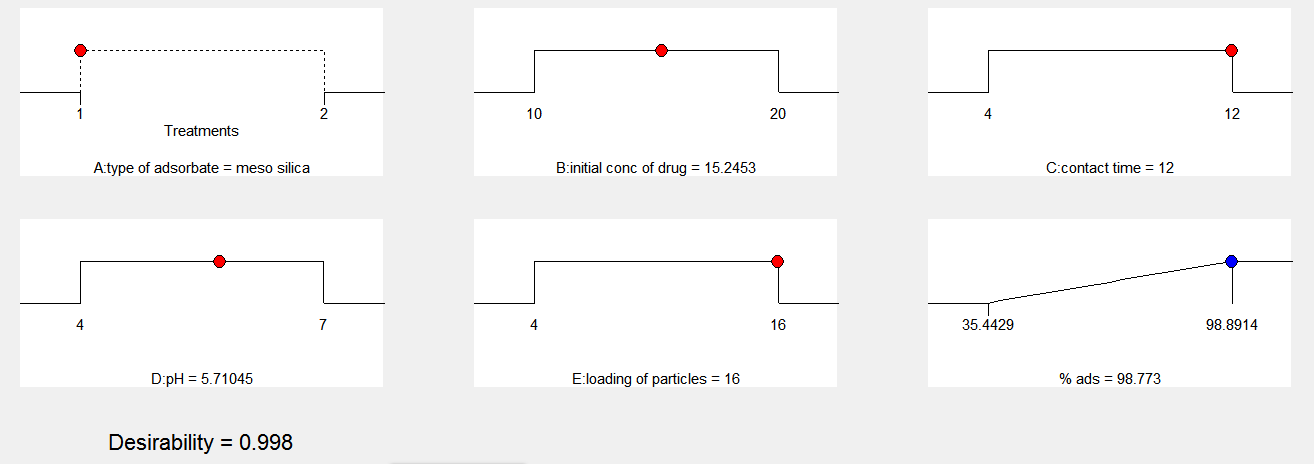


**b**

Fig. S3. The optimum conditions (showing the factors values for pH, initial drug concentration, contact time and the MSNP concentration) for the maximum adsorption of LEVO by (a) MPNP, (b) MSNP

**Fig. S4. Langmuir adsorption isotherm and data of Levofloxacin adsorption to MPNP.**

**Fig. S5. Frendlich adsorption isotherm and data of Levofloxacin adsorption to MPNP.**

**Fig. S6. Redlich-Peterson adsorption isotherm and data of** **Levofloxacin adsorption to MPNP.**

**Fig. S7. Sips adsorption isotherm and data of Levofloxacin adsorption to MPNP.**

**Table S1. AGREE reports of the proposed procedures.**


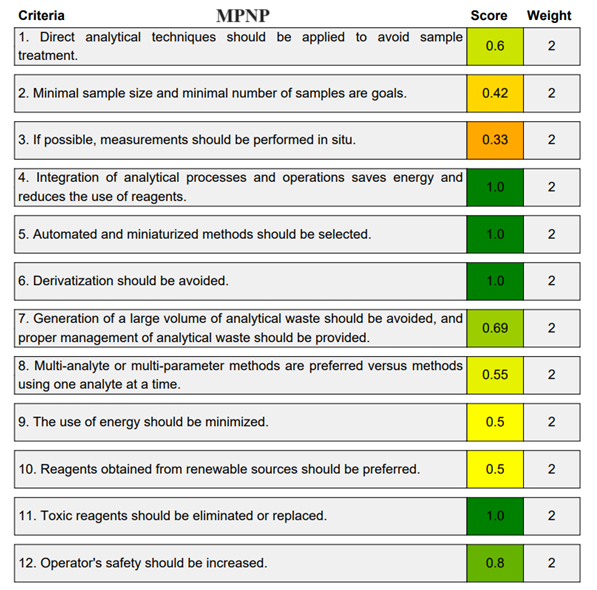


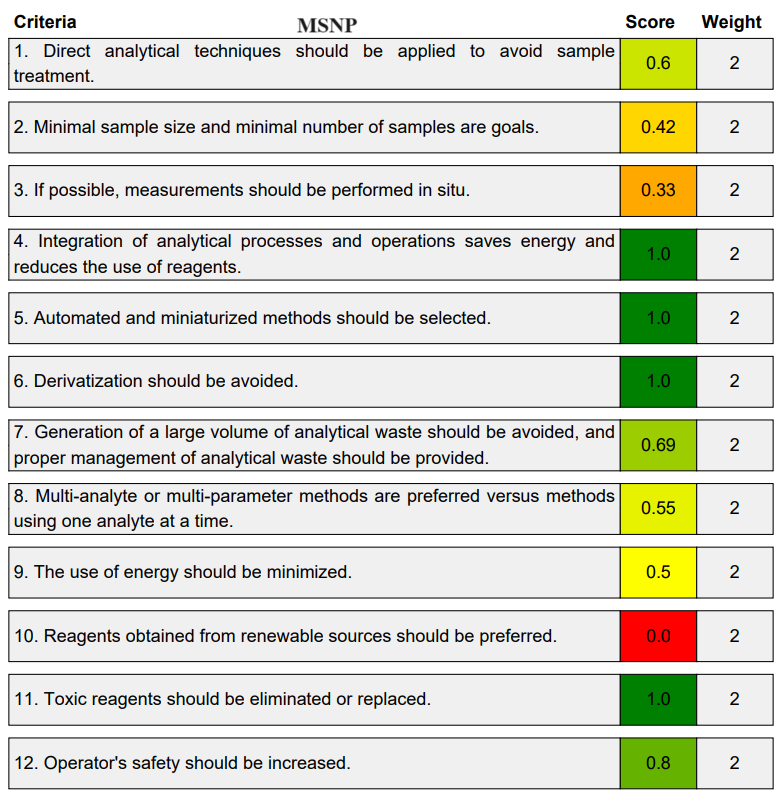


**Table S2. Figures of Merit 1 (FM-1) of Hexagon-CALIFICAMET.**

|  | | **Penalty points (Pps)** |
| --- | --- | --- |
| **Sample treatment and preparation** | Preservation | 0 |
|  | Storage | 0 |
|  | Amount | 1 |
|  | Reagents / solvents used | 1 |
|  | Amount of reagents, solvents | 2 |
|  | Instrumental vs Adequacy to the method | 0 |
|  | Number of weekly samples | 1 |
|  | Pretreatment | 0 |
|  | **Σ Pps** | **5** |
| **Method characteristics** | Method categories | 1 |
|  | Operational mode | 0 |
|  | Portability | 0 |
|  | Method / sample | 0 |
|  | Analytes/ sample | 1 |
|  | Time of analysis/sample | 2 |
|  | Robustness | 0 |
|  | **Σ Pps** | **4** |
| **Calibration** | Frequency | 2 |
|  | Required time | 2 |
|  | Number of standards | 2 |
|  | Lineal adjustment R^2^ | 0 |
|  | LOD, LOQ limits | 2 |
|  | Working range and linearity | 0 |
|  | Precision | 1 |
|  | **Σ Pps** | **9** |

**Table S3. Figures of Merit 2 (FM-2), toxicity and safety of Hexagon-CALIFICAMET.**

|  | | **Penalty points (Pps)** |
| --- | --- | --- |
| **Quality control** | Frequency | 1 |
|  | Time required | 3 |
|  | Number of standards | 1 |
|  | **Σ Pps** | **5** |
| **Accuracy** | Frequency | 1 |
|  | Time required | 3 |
|  | Concentration levels | 1 |
|  | Magnitude / Size | 0 |
|  | Selectivity | 0 |
|  | **Σ Pps** | **5** |
| **Toxicity: health and environmental hazards** | Severe toxicity | 3 |
|  | Corrosive | 0 |
|  | Irritating | 0 |
|  | Irritating to eyes | 2 |
|  | Irritating to the respiratory system and skin | 3 |
|  | Mutant | 3 |
|  | Carcinogen | 3 |
|  | Toxic/Poisonous | 3 |
|  | Systemic toxicity for target organ | 3 |
|  | Toxicity to the aquatic environment | 0 |
|  | Amount of reagent | 3 |
|  | **Σ Pps** | **23** |
| **Safety: Physical hazards** | Explosives | 0 |
|  | Flammables | 1 |
|  | Causes burns | 0 |
|  | Low-pressure gases | 0 |
|  | Self-reactive substances | 0 |
|  | Pyrophorics | 1 |
|  | Substances experience spontaneous heating | 0 |
|  | Water-activated flammable gases | 0 |
|  | Organic peroxides | 0 |
|  | Corrosive for metals | 0 |
|  | Amount of reagent | 3 |
|  | **Σ Pps** | **5** |

**Table S4. Penalty points (PPs) to assess waste generation and annual economic cost of Hexagon-CALIFICAMET**

|  | | **Penalty points (Pps)** |
| --- | --- | --- |
| **Waste generation** | Amount | 3 |
|  | Waste treatment | 0 |
|  | Disposal material | 2 |
|  | **Σ Pps** | **5** |
| **Economic cost** | Number of samples | 2000 |
|  | Time of analysis (hours) | 4 |
|  | Equipment cost | 100 |
|  | Amortization period | 10 |
|  | Salary skilled personnel | 200 |
|  | Electricity consumption | 100 |
|  | Reagents | 50 |
|  | Consumable materials | 50 |
|  | **Σ Pps** | 2514 |

**Table S5. Overall qualification (OQ) of the variables of the method according to penalty points ranges.**

| **Variable** | **Σ Pps** | **OQ** |
| --- | --- | --- |
| FM-1 | 18 | 2 |
| FM-2 | 10 | 2 |
| Toxicity | 23 | 3 |
| Safety | 5 | 1 |
| Residues | 5 | 0 |
| Carbon footprint | 1.78 | 3 |
| Economic cost | 2514 | 0 |
